# Interfacial Engineering of Nickel Oxide‐Perovskite Interface with Amino Acid Complexed NiO to Improve Perovskite Solar Cell Performance

**DOI:** 10.1002/smll.202405953

**Published:** 2024-09-20

**Authors:** Dilpreet Singh Mann, Sakshi Thakur, Sushil S. Sangale, Kwang‐Un Jeong, Sung‐Nam Kwon, Seok‐In Na

**Affiliations:** ^1^ Department of Flexible and Printable Electronics and LANL‐JBNU Engineering Institute‐Korea Jeonbuk National University 567, Baekje‐daero, Deokjin‐gu Jeonju‐si 54896 Republic of Korea; ^2^ Department of Polymer‐Nano Science and Technology Department of Nano Convergence Engineering Jeonbuk National University 567, Baekje‐daero, Deokjin‐gu Jeonju‐si 54896 Republic of Korea

**Keywords:** charge carrier dynamic, low temperature, NiO complex, perovskite solar cells, redox reaction

## Abstract

The interface between NiO and perovskite in inverted perovskite solar cells (PSCs) is a major factor that can limit device performance due to defects and inappropriate redox reactions, which cause nonradiative recombination and decrease in open‐circuit voltage (VOC). In the present study, a novel approach is used for the first time, where an amino acid (glycine (Gly), alanine (Ala), and aminobutyric acid (ABA))‐complexed NiO are used as interface modifiers to eliminate defect sites and hydroxyl groups from the surface of NiO. The Ala‐complexed NiO suppresses interfacial non‐radiative recombination, improves the perovskite layer quality and better energy band alignment with the perovskite, resulting in improved charge transfer and reduced recombination. The incorporation of the Ala‐complexed NiO leads to a PCE of 20.27% with enhanced stability under the conditions of ambient air, light soaking, and heating to 85 °C, as it retains over 82%, 85%, and 61% of its initial PCE after 1000, 500, and 350 h, respectively. The low‐temperature technique also leads to the fabrication of a NiO thin film that is suitable for flexible PSCs. The Ala‐complexed NiO is fabricated on the flexible substrate and achieved 17.12% efficiency while retaining 71% of initial PCE after 5,000 bending.

## Graphical abstract

In this work, the Ala‐complexed NiO suppresses interfacial non‐radiative recombination, improves the perovskite layer quality, and provides more favorable energy band alignment with the perovskite, resulting in improved charge transfer and reduced recombination. The incorporation of the Ala‐complexed NiO leads to a power conversion efficiency (PCE) of 20.27% with enhanced stability under various conditions.

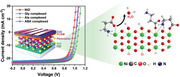

## 1 Introduction

Organometal halide perovskites have excellent optoelectronic features, including a precisely adjusted energy band gap, low exciton energy, good carrier transport, high charge diffusion length, and high resistance to defects, that make them useful in many optoelectronic fields.^[^ ^1^, ^2^, ^3^, ^4^, ^5^ ^]^ Over the past few years, the power conversion efficiency (PCE) of perovskite solar cells (PSCs) has reached 26.4%, which has been achieved with better thin‐film growth, interface, and absorber materials.^[^ ^5^, ^6^ ^]^ However, the major barrier to the commercialization of PSCs is stability restriction, which occurs due to degradation issues of perovskites.^[^ ^7^, ^8^, ^9^ ^]^ To overcome this issue, the past few years have seen significant interest in inverted (p‐i‐n) perovskite solar cells (PSCs) because of their ability to be processed at low temperatures, their improved compatibility with flexible substrates, and their suitability for tandem device production.^[^ ^10^, ^11^ ^]^ Nickel oxide (NiO) is considered to be a promising hole‐transport layer (HTL) in inverted perovskite solar cells (PSCs) because of its large band gap (3.6–4.0 eV), high transmittance, and high chemical stability.^[^ ^12^, ^13^ ^]^ Additional benefits of NiO include its affordable price, abundance in the crust, and easy and cost‐efficient synthesis.^[^ ^14^, ^15^, ^16^, ^17^ ^]^

However, the reliability and efficiency of these NiO‐based PSCs are still inadequate due to interfacial loss and deterioration. The presence of chemically reactive species such as NiOOH and Ni(OH)_2_ can lead to the degradation of perovskite layers by producing interface energy barriers and redox reactions when interacting with organic compounds in the perovskite solution, such as methylammonium iodide (MAI) and formamidinium iodide (FAI).^[^ ^18^, ^19^ ^]^ The decrease in high‐oxidation‐state Ni species (Ni^3+^/Ni^2+^ ratio) of NiO leads to poor electrical conductivity, which results in reduced hole extraction and transportation.^[^ ^20^, ^21^ ^]^ The perovskite layer coated onto the HTL also exhibits poor wettability on the NiO films, which reduces the grain size and poor crystal structure of perovskite, resulting in poor charge transportation and extraction between the perovskite and NiO heterojunction interface.^[^ ^22^, ^23^ ^]^ Further, NiO thin films possessing a low valance band (VB) state demonstrate inappropriate band alignment with the perovskite layer, which leads to decreased open‐circuit voltage (V_OC_) and fill factor (FF).^[^ ^24^, ^25^ ^]^ These factors make it more difficult to achieve high PCE and long‐term operational stability during the manufacturing process. Consequently, employing advanced technologies to develop the interface of NiO is considered to be a feasible approach to enhancing the efficiency of PSCs.

Several methods have been used to fix these defects and suppress non‐radiative recombination at the interface between NiO and perovskite.^[^ ^19^, ^26^, ^27^ ^]^ Incorporating metal ions into the NiO layer through doping is a viable approach to enhancing its conductivity, thereby facilitating hole extraction and impeding charge recombination.^[^ ^19^, ^28^, ^29^ ^]^ In contrast, adding more metal ions might lead to the creation of trap states as well as interface losses from surface defect sites, and an inability to eliminate the hydroxyl groups on the NiO surface.^[^ ^30^ ^]^ Surface modification could also be an effective and versatile passivation method that involves adding certain materials between the NiO and perovskite layers.^[^ ^19^, ^26^ ^]^ Small organic compounds, such as amine and their salts, are often used to modify the interface. These compounds can improve energy level alignment between the NiO and perovskite layers and ultimately achieve high conductivity of the NiO layer, which leads to improved charge transfer and reduction in the recombination process.^[^ ^31^, ^32^ ^]^ Several small molecules containing various functional groups also show the ability to control the nucleation and crystallization processes of perovskite films, thereby resulting in efficiently neutralizing hidden interface defects.^[^ ^33^, ^34^ ^]^ For instance, McGehee et al. used an excessive number of A‐site cation salts as surface agents to stop the formation of a PbI_2‐x_Br_x_ layer that is caused by Ni^>3+^, which stopped voltage loss and made the material more stable over time at high temperatures.^[^ ^19^ ^]^ In another study, Wu et al. reported that the addition of the hybrid interfacial layer made of SAM (trimethyl sulfonium bromide (TMSBr)) may improve the interface of NiO energy level alignment, charge extraction, and chemical stability, leading to great PCE.^[^ ^18^ ^]^ Wang et. al. studied an interfacial modification strategy with a multi‐fluorine organic molecule 6FPPY, which is proposed to manage the interface of NiO, which passivate the NiO/perovskite interface defects, and suppresses the detrimental reaction between NiO and perovskite and achieve the champion PCE of 24.0%.^[^ ^35^ ^]^ Further, many additional materials, such as organic polymers, inorganic thin films, and carbon quantum dots, are used to passivate interfacial defects, which also demonstrates significant efficacy in surface passivation of NiO films.^[^ ^36^, ^37^, ^38^ ^]^ However, the focus of these investigations mostly revolves around two methods: first, reducing the NiO defects, and second, including an interlayer to prevent undesirable interface chemistry. However, both of these methods result in elevated fabrication complexity due to the introduction of an extra interlayer,^[^ ^39^ ^]^ and there have been no significant efforts to adjust the reactive nature of Ni^3+^ ions and the reduction of the hydroxyl group by specifically focusing on modifying the nickel oxide HTL in bulk.^[^ ^39^, ^40^, ^41^ ^]^

Recent studies have resolved the interfacial contact between metal oxide and perovskite issues using a new unique approach, where the complex metal oxide is synthesized using small molecules. In a recent study on PSCs, Yang et al. established a method for developing EDTA‐complexed SnO_2_ (ESnO_2_) electron transport layers (ETLs), which led to improved grain size, reduced trap density, and enhanced crystallinity, which facilitated better hole extraction and transport, as well as mitigated perovskite degradation at grain boundaries.^[^ ^42^ ^]^ Further, Wu et al. added carboxyl (COOH) terminal ligands evenly throughout the metal oxide ETL to make continuous pathways for electron transport, which can improve electron mobility and energy level alignment.^[^ ^43^ ^]^ The results of these studies show that complex metal oxides could be a very useful way to change the interface while providing great electrical and optical properties as well as chemical stability. In this study, we demonstrated that the amino acid‐based complex can be successfully synthesized with NiO and used as the HTL in PSCs. The formation of a complex by the interaction of an amino acid and a transition metal oxide is a well‐established and reported phenomenon.^[^ ^44^, ^45^ ^]^ The complex formation ability arises from the capability of amino acid to donate its lone‐pair electrons to the unoccupied d‐orbital of the transition metal atom.^[^ ^46^ ^]^ Therefore, amino was selected to form complexes with the entire NiO particles, with the aim of eliminating surface defects and the hydroxyl group, thus improving the optical and electrical properties of the NiO. In addition, these glycine (Gyl), alanine (Ala), and aminobutyric acid (ABA) amino acids have unique structures and functionality compared to the other amino acids. These amino acids exhibit different functional groups (COOH and NH_3_) at the side chain (instead of carbon as is the case with all other amino acids). The presence of different functional groups and their Lewis basicity can affect the bonding capability of the molecule to form complexes with electron‐deficient molecules that are better than other amino acids.^[^ ^47^ ^]^ We conducted a comprehensive analysis of three amino acid molecules, including glycine (Gyl), alanine (Ala), and aminobutyric acid (ABA), with varying chain lengths to determine the optimal complex formation to develop superior materials for interfacial modification. With the increase in the chain length of the molecule, the conformational flexibility of molecules is improved, which leads the better bonding strength and interaction ability. Further, properties of amino acids are enhanced with changes in chain length such as reaction rate, hydrophobic nature, and bonding strength.^[^ ^48^, ^49^, ^50^ ^]^ In the novel approach used for the first time here, an amino acid complexed NiO was utilized as an interface modifier to eliminate defect sites and hydroxyl groups from the surface of NiO. As a result, the amino acid complexed NiO successfully resolves various problems by neutralizing interfacial defects, reducing nonradiative recombination, and inhibiting unappropriated redox reactions at the NiO/perovskite interface. These outcomes ultimately lead to significantly improved V_OC_ and PCE of the devices. Furthermore, the presence of amino acid complexed NiO also resulted in improved alignment of the energy levels between the NiO HTL and the perovskite layer. This led to more efficient extraction and transport of charges, while the occurrence of charge recombination was also reduced. Moreover, the amino acid complexed NiO increased the crystal structure and grain size of the perovskite due to the high contact angle, which resulted in greater charge extraction and transportation. The device utilizing Ala‐complexed NiO achieves a noteworthy FF of 80.15%, a short‐circuit current density (J_SC_) of 22.37 mA cm^−2^, a high V_OC_ of 1.13V, and a champion PCE of 20.27%. Ala complexed NiO PSCs demonstrate enhanced stability while exposed to the conditions of ambient air, continuous light irradiation, and heating to 85 °C, maintained over 82%, 85%, and 61% of their original PCE after 1000, 500, and 330 h, respectively. Further, the low‐temperature NiO deposition technique is beneficial for fabricating flexible PSCs. Therefore, Ala‐complexed NiO flexible PSCs have successfully fabricated, achieved an efficiency of 17.12%, and retained 71% of their original PCE even after completing 5 000 bending tests.

## 2 Results and Discussion

NiO powder was synthesized using a simple co‐precipitation method.^[^ ^25^, ^51^ ^]^ The synthesis procedure is detailed in the supporting information. The phase identification of the synthesized NiO powder was conducted through X‐ray diffraction (XRD) analysis. The sample has large diffraction bands, as shown in Figure S1a (Supporting Information). The diffraction peaks of NiO, which are at 2θ = 36.53°, 42.73°, and 62.04°, respectively correspond to the (111), (200), and (220) planes of NiO with exhibits of the face‐centered cubic (FCC) crystal system.^[^ ^25^ ^]^ These peaks confirm the formation of the NiO NPs and are well‐matched with the Joint Committee on Powder Diffraction Standard (JCPDS) card no. 03‐065‐2901.^[^ ^51^ ^]^ Moreover, the resultant dark‐black powder also exhibits excellent dispersion in DI water even after 30 days, as shown in Figure S1b (Supporting Information). Further, Figure S2a (Supporting Information) depicts the transmission spectra of the NiO films. The average optical transmittance of the nickel oxide nanoparticles (NiO NPs) thin films achieves 91% over the wavelength range from 400 to 800 nm. The high transmittance of the NiO films indicates that the presence of NiO NPs leads to minimal optical losses along with efficient production of photo‐induced carriers in the PSCs. The band gap (E_g_) of the NiO films was also investigated by analyzing the Tauc plots, as illustrated in Figure S2b (Supporting Information). By assuming a direct transition between the conduction and valence bands, the optical E_g_ can be calculated from the plot using the following equation:^[^ ^25^ ^]^

(1) $$\alpha h\nu =A{\left(h\nu -E_{g}\right)}^{n}$$

where α represents the absorption coefficient, A indicates a proportionality constant, h designates Planck's constant, ν represents the frequency of the incident photon, and n = 1/2 depicts a direct transition.^[^ ^52^ ^]^ The calculated E_g_ of the NiO NPs is approximately 3.97 eV, which is similar to the corresponding value reported in the previous study.^[^ ^53^, ^54^, ^55^ ^]^ This suggests that the synthesized NiO has a wider band gap, which enhances its potential for hole extraction and electron‐blocking ability.^[^ ^53^, ^54^, ^55^ ^]^ These observations demonstrated that NiO was effectively synthesized using a coprecipitation technique at a lower annealing temperature (270 °C) without the need to use surfactants or capping agents,^[^ ^25^, ^51^, ^56^ ^]^ and NiO exhibited a high value of E_g_, which shows its high potential as a material for hole extraction and electron‐blocking.

**Figure 1:**
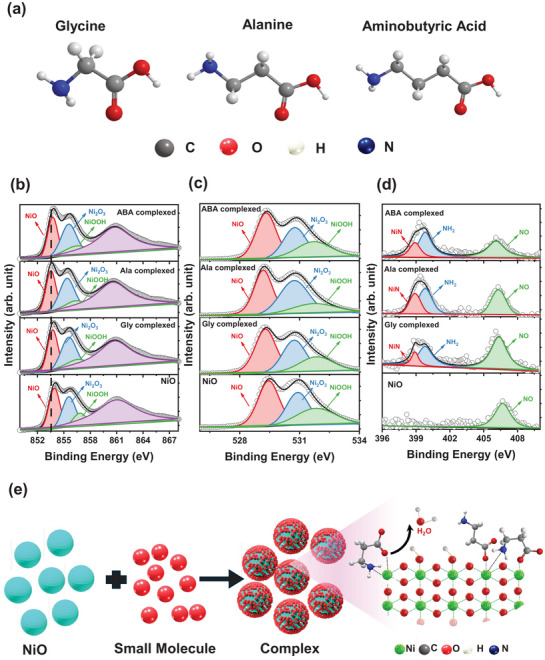
a) Schematic illustration of various amino acid molecule structures, high‐resolution X‐ray photoelectron spectroscopy (XPS) spectra of b) Ni 2p, c) O 1s, and d) N1s of amino acid complexed NiO thin films, and e) schematic illustration of the mechanism of amino acid complex formation by eliminating the hydroxyl groups on the surface of NiO.

Further, to systematically investigate the interaction and formation of the complex with synthesis NiO NPs with amino acid molecules, three amino acid‐based molecules were selected: glycine, alanine, and aminobutyl acid, which consist of similar carboxyl and amine functional groups with different alkane chain lengths, as shown in **Figure** **1**a. In addition, we also conducted an in‐depth investigation using high‐resolution X‐ray photoelectron spectroscopy (XPS) to forecast the impact of the chemical interaction between different amino acids with NiO on the surface chemistry as well as the oxidation states of nickel. Figure 1b,c,d displays the characteristic XPS spectra of the core states (Ni 2p, O 1s, and N 1s) for NiO and different amino acid complexed NiO thin films. Figure 1b shows that the Ni 2p spectra of NiO and different amino acid complexed NiO have four different peaks. The satellite peak observed at 860.5 eV (purple) corresponds to the shaking mechanism occurring in the typical NiO structure.^[^ ^57^ ^]^ At the same time, the peak at 853.5 eV is assigned to NiO (Ni^2+^, red) because of its octahedral bonding structure.^[^ ^58^ ^]^ The peak at 855.4 eV is assigned to Ni_2_O_3_ (Ni^3+^, blue) because it has a Ni^2+^ vacancy.^[^ ^59^ ^]^ The 856.5 eV peak, which is assigned to NiOOH (β‐NiOOH or γ‐NiOOH).^[^ ^18^, ^19^ ^]^ Further, the O1s spectra analysis indicated the presence of three distinct chemical species: NiO (Ni^2+^, red), Ni_2_O_3_ (Ni^3+^, blue), and NiOOH (Ni^≥3+^, green), which were observed at the energy levels of 529.1, 530.7, and 531.8 eV, respectively, as depicted in Figure 1c.^[^ ^24^, ^36^ ^]^ Consequently, the notable rise in the N 1 s signal complexation with amino acid, as depicted in Figure 1d, suggests the existence of nitrogen species formed through the chemical interaction between the amino acid molecule and NiO. The N1s spectra of the amino acid complexed NiO exhibit NO signals at ≈406.7 eV, NH_2_ signals at ≈399.8 eV, and NiN signals at ≈398.8 eV, indicating the presence of amino acid in the NiO thin film, and the NH_2_ functional group of amino acid interacts with Ni of NiO and forms Ni─N bonding.^[^ ^26^, ^60^, ^61^, ^62^, ^63^, ^64^ ^]^ However, NO signals were also observed at ≈406.7 eV in the pristine NiO thin film. This peak could be attributed to gas‐phase NO molecules due to the chemical environment around the nitrogen atoms during the measurement.^[^ ^65^, ^66^, ^67^ ^]^ Further, the proportions of nickel oxide species in the Ni2p and O1s spectra that include NiOOH have been reduced in the amino acid complexed NiO compared to the unmodified NiO, as illustrated in Figure S3a (Supporting Information). This suggests that the amino acid complexed NiO effectively removes the defect sites and hydroxyl groups that are present on the surface of the NiO NPs thin film. Moreover, Figure S3b (Supporting Information) displays the Ni_2_O_3_/NiO (Ni^3+^/Ni^2+^) ratio calculated from the XPS spectra of Ni 2p and O1s, which indicates that the amino acid‐complexed NiO film exhibits a higher ratio of Ni^3+^/Ni^2+^ due to the higher concentration of Ni^3+^, which results from the interaction between the amino acid and NiO.^[^ ^68^ ^]^ The higher Ni^3+^/Ni^2+^ ratio of the amino acid‐complexed NiO could result in improved conductivity,^[^ ^62^, ^69^ ^]^ because raising the concentration of Ni^3+^ can encourage the production of more holes.^[^ ^70^, ^71^ ^]^ The p‐type characteristics of the nickel oxide layer can also be caused by the high concentration of Ni^3+^.^[^ ^57^, ^71^ ^]^ It can also clearly be seen that, in the amino acid‐based complexed NiO, Ni^3+^ chemical species were increased while NiOOH chemical species decreased, which indicates that the complexation led to a reduction in the hydroxyl group and surface defect sites from the NiO NPs and that superior charge transfer properties can also be achieved.^[^ ^19^, ^62^ ^]^ The XPS results suggest that the Ni 2p peaks of the amino acid‐based complex exhibited a shift toward lower binding energy compared to NiO. The shift in binding energy observed in XPS spectra can be attributed to the varying electronegativities of metal ions or the strong interaction, which involves electron transfer between materials.^[^ ^72^, ^73^ ^]^ The shifting toward lower binding energy indicates the latter phenomenon, which is a strong interaction, as this leads to the formation of Ni─O─C bonds at the interface between NiO and the amino acid molecule, which confirms the formation of the amino acid complexed NiO.^[^ ^26^, ^73^ ^]^ These observations demonstrate that the H of the carboxyl functional group of amino acids interacts with a defect site (OH) on the surface of NiO, resulting in the formation of Ni─O─C bonds and the release of H_2_O as a byproduct, and the NH_2_ functional group of amino acids donates the lone pair to Ni and establishes the coordination bond with the Ni ion of NiO, which leads to the formation of the Ni─N bond.^[^ ^26^, ^62^, ^74^, ^75^ ^]^ This process leads to the formation of a complex with NiO, as shown in Figure 1e. Further, the formation of a complex with amino acid materials results in increased percentages of nitrogen and carbon in the amino acid‐based complexes compared to pristine NiO, as shown in Figure S3c (Supporting Information). Here, the Ala complexed NiO exhibits higher percentages of nitrogen compared to other amino acids‐based complexed NiO, which is likely attributable to the increased interaction with NiO compared to the other amino acid complexed NiO.^[^ ^26^ ^]^ The Ala‐complexed NiO also displayed a more favorable shift toward lower energy in both the XPS spectra. This was accompanied by an improvement in the Ni^3+^/Ni^2+^ ratio and a reduction in the hydroxyl group compared to the other complex. These results suggest that alanine can show a stronger interaction with NiO than the other amino acid complexed NiO. These findings demonstrate that the successful formation of Ala complexed NiO thin film and Ala complexed NiO showed promising interactions with NiO compared to the other amino acid complexed NiO, which led to a rise in the ratio of Ni^3+^ to Ni^2+^ and eliminated the hydroxyl group, resulting in enhanced hole conductivity and reduction of the defect site in the Ala complexed NiO.

**Figure 2:**
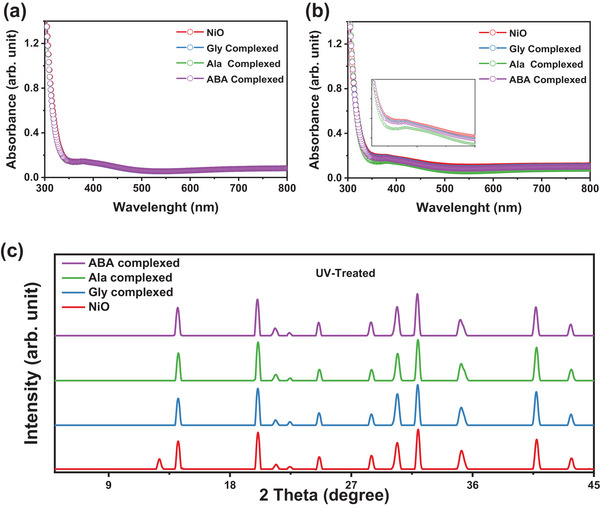
UV–vis spectra of a) fresh and b) UV‐treated NiO and amino acid complexed NiO thin films, and c) XRD patterns of the perovskite layer coated on the UV‐treated NiO and amino acid complexed NiO.

Furthermore, the redox reaction between the Ni^≥3+^ and hydroxyl groups in the NiO and perovskite layer results in oxidized CH_3_NH_3_^+^, which reacts with PbI in the perovskite. The hydroxyl group on the NiO surface, specifically the OH, can readily react with the CH_3_NH_3_^+^ ion and PbI to produce CH_3_NH_2_, H_2_O, and PbO, respectively, resulting in perovskite degradation.^[^ ^18^, ^62^ ^]^ Therefore, the effect of the hydroxyl group of the various amino acid molecules to form a complex with NiO was also confirmed using UV–vis spectroscopy and X‐ray diffraction spectroscopy (XRD). **Figure** **2**a shows that both NiO and the amino acid complexed NiO exhibit similar optical transmittance, suggesting that the complex does not affect the optical properties of the NiO thin film. Further, to confirm and evaluate the effective use of the NiO complex, we evaluated UV‐ozone (UV)‐treated NiO NPs thin films and NiO complex thin films formed by various amino acids, because the UV light generated more hydroxyl groups on the NiO surface, which leads to a reduction in the absorbance properties of the NiO.^[^ ^19^ ^]^ Consistent with the results of prior studies, the UV–vis absorption spectra of UV‐treated NiO showed high absorbance compared to the spectra of without UV‐treated NiO, which indicates an increase in sub‐bandgap absorption (Figure 2b).^[^ ^62^ ^]^ Moreover, the optical absorbance of the amino acid complexed NiO thin film was comparable to that of the pure NiO NPs thin film without UV‐treated samples and amino acid complexed NiO (Figure 2b). Considering that the difference in absorption has been attributed to an increase in NiOOH defect sites, it can be concluded that the amino acid complex formation effectively interacts with the NiO, which could reduce the surface defect density.^[^ ^19^, ^62^ ^]^ In addition, the Ala complexed NiO also showed a low absorbance compared to the other amino acid complexed NiO, which could indicate that the interaction between the alanine and the NiO is better than that of other amino acid molecules, as shown in the inset of Figure 2b. The effect of the hydroxyl group of the NiO surface on the perovskite layer was further confirmed through X‐ray diffraction spectroscopy. The X‐ray diffractogram of the UV‐treated NiO NPs reveals the presence of the small PbI_2−x_Br_x_ phase at a peak position of 12.8°, and the intensity of the (001) peak is reduced, as can be seen in Figure 2c. The degradation of the (001) peak in the perovskite layer coincides with the appearance of the PbI_2‐x_Br_x_ peak.^[^ ^76^, ^77^ ^]^ The presence of PbI_2‐x_Br_x_ and the reduction of the (001) peak are attributed to the lack of the A‐site cations, which leads to rapid degradation in the PSCs performance, which is attributed to the reductions in charge extraction and transportation capability with high recombination rate.^[^ ^19^, ^26^ ^]^ Furthermore, the lack of A‐site cations within the perovskite layer can also impact the arrangement of crystallographic planes, which causes degradation in the perovskite crystal structure.^[^ ^19^ ^]^ However, the XRD pattern of the UV‐treated amino acid complexed NiO did not show any PbI_2−x_Br_x_ phase. The results emphasize that the amino acid complexed NiO functions as a restorative intermediary, effectively minimizing defects like Ni^≥3+^ species or hydroxyl groups on the different NiO surfaces.^[^ ^19^, ^62^ ^]^ These results indicate that the Ala complexed NiO is more effective in preventing the redox reaction at the NiO/perovskite interface than other amino acid complexed NiO and pure NiO, which is attributed to its superior ability to reduce defect sites and hydroxyl groups on the surface of the NiO NPs thin film, which inhibits the degradation of perovskites through redox reactions and improves charge transfer characteristics.^[^ ^18^, ^62^ ^]^

**Figure 3:**
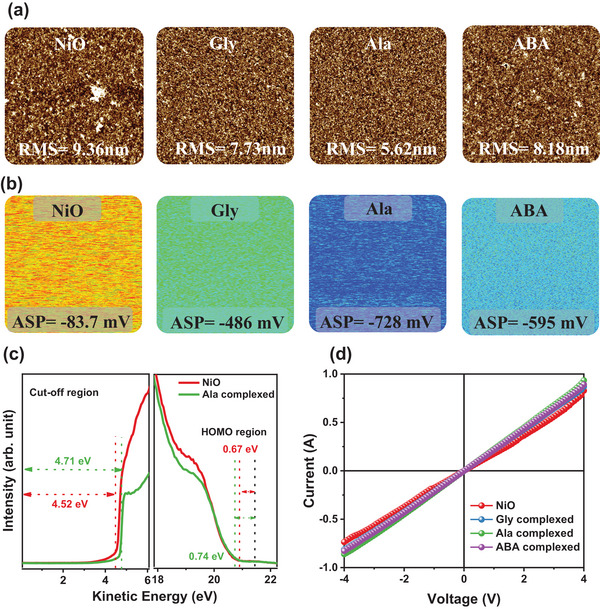
a) Atomic force microscopy (AFM), b) scanning kelvin probe microscopy (SKPM) images of NiO and amino acid complexed NiO thin films (where ASP is average surface potential), c) UPS spectra of NiO and Ala complexed NiO, and d) *I–V* curve of the NiO and amino acid complexed NiO.

To evaluate alterations in the physical and electrical characteristics of the NiO NPs thin film resulting from the amino acid complexed NiO, we conducted investigations using atomic force microscopy (AFM), scanning Kelvin probe microscopy (SKPM), and ultraviolet‐visible spectroscopy (UPS). The physical surface characteristics of the thin films of pure NiO NPs and amino acid complexed NiO showed very little difference. Figure S4 (Supporting Information) showed the SEM images of pure NiO, Gly, Ala, and ABA complexed NiO thin film. The pristine NiO thin film showed the non‐uniform coverage and a lot of the defect site on the surface, as shown in Figure S. In addition, amino acid complexed NiO showed a uniform and smoother thin film compared to the pristine NiO. Furthermore, to check the more detailed surface condition of the pure NiO, Gly, Ala, and ABA complexed NiO thin film, the AFM was measured as shown in **Figure** **3**a. As observed in Figure 3a, the surface roughness of the film changes depending on the complexation of the NiO layer: the unmodified NiO layer and the Gly, Ala, and ABA complexed NiO are 10.36, 7.73, 5.62, and 8.18 nm, respectively. The smoother surface of complex NiO could provide better hole extraction and reduce recombination.^[^ ^26^, ^78^ ^]^ The electrical properties of the surface also exhibited a notable alteration with the amino acid complexed NiO by SKPM, as can be seen in Figure 3b. The thin film of Ala complexed NiO exhibited a surface potential value of −728 mV, which was lower than the surface potential values of the pristine NiO NPs thin film (−83.7 mV), Gly complexed NiO based thin film (−486mV), and ABA complexed NiO based thin film (−595 mV). This suggests that the Ala complexed NiO has a higher work function (WF) than the pristine NiO and other amino acid complexed NiO.^[^ ^79^, ^80^ ^]^ The obtained results were very congruent with the UPS analysis results (Figure 3c). As shown in Figure 3c, the valence band maximum (VBM) of the Ala complexed NiO film increases from 5.19 to 5.45 eV, while the work function (WF) increases from 4.52 to 4.71 eV. As shown in Figure S5a (Supporting Information), the VBM of Gly complexed NiO and ABA complexed NiO are 5.29 and 5.35 eV, respectively, while their WF values are 4.58 and 4.64 eV, respectively. The VBM offsets for pristine NiO, Gly, Ala, and ABA complexed NiO are 0.52, 0.42, 0.26, and 0.36 eV, respectively. Based on these findings, the band diagram depicted in Figure S5b (Supporting Information) indicates that the Ala complexed NiO possesses an energy level that is appropriate for perovskite layers. These results indicate that the Ala complexed NiO possesses a more p‐type characteristic compared to pure NiO and other amino acid complexed NiO.^[^ ^24^, ^62^ ^]^ The deeper shifting of the work function and VBM of the Ala complexed NiO could be due to the fact that the alanine molecules can better interact with NiO than the other amino acid complexed NiO, which leads to the increased presence of high‐content of nitrogen atoms in complexed NiO to have higher electronegativity, which can cause a deeper work function and VBM shift with alanine complexed NiO compared to other amino acid complexed NiO.^[^ ^23^, ^26^, ^62^, ^81^ ^]^ To examine the ability to extract holes and the conductivity, the current–voltage (*I‐V*) characteristics of the device were assessed as shown in Figure 3d. The electrical conductivity of the NiO and amino acid complexed NiO films are determined by applying the following equation:

(2) $$I=\frac{\sigma_{0}A}{L}$$

where A represents the area of the device, while L denotes the thickness of the film. The conductivity values for NiO and the Gly, Ala, and ABA complexed NiO are 3.15 ×  10^−4^ S cm^−1^, 4.32  × 10^−4^ S cm^−1^, 5.79 × 10^−4^ S cm^−1^, and 4.85 × 10^−4^ S cm^−1^, respectively. The Ala complexed NiO has been noted to have a greater current density compared to both the pure NiO and other amino acid complexed NiO. This suggests that the Ala complexed NiO has a superior capacity to extract holes relative to the pristine NiO and other amino acid complexed NiO.^[^ ^25^, ^69^ ^]^ This is consistent with the earlier XPS findings, which point to an enhancement in electrical conductivity as a result of an increase in the Ni^3+^/Ni^2+^ ratio following alanine complexation.

**Figure 4:**
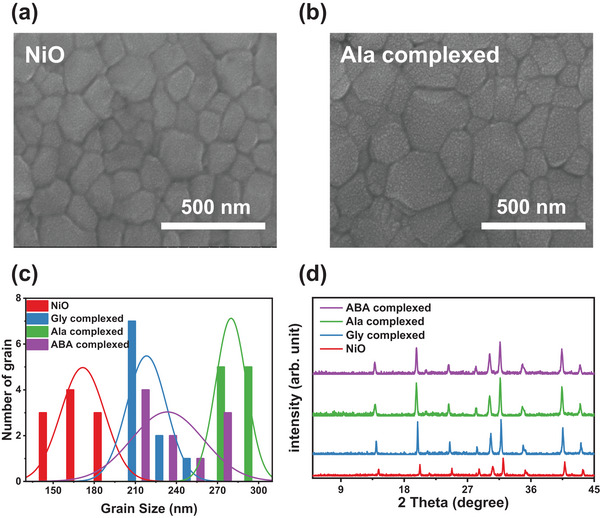
SEM images of perovskite films fabricated onto (a) NiO and (b) Ala complexed NiO, (c) statistical diagrams of grain size distribution based on SEM images, and (d) XRD spectra of NiO and amino acid complexed NiO.

To confirm the impact of an amino acid complexed NiO film on the grain size and crystallinity of perovskite, SEM, contact angle (CA), and XRD analyses were conducted on perovskite layers that were deposited onto pristine NiO, Gly, Ala, and ABA complexed NiO thin layers. **Figures** **4**a,b and S6 (Supporting Information) show the SEM images of the surface of pure NiO, Gly, Ala, and ABA complexed NiO with perovskite layers, respectively. The enhancement in the grain size of the perovskite layer deposited on the NiO complex thin film was more notable than that of the perovskite layer deposited on the NiO thin film. Specifically, the grain size histogram in Figure 4c indicates that the perovskite film coated over an Ala complexed NiO has a wider range of grain sizes, with an average size of 298 nm, when compared to pure NiO and other amino acid complexed NiO. This assertion is substantiated by the contact angle (CA) measurements of pure NiO, Gly, Ala, and ABA complexed NiO thin layers, depicted in Figure S7 (Supporting Information). The hydrophobicity of the Ala complexed NiO (with a contact angle of ≈32.16°) was greater than that of the Gly complexed NiO (with a contact angle of 21.76°), the ABA complexed NiO (with a contact angle of 27.17°), and NiO (with a contact angle of 10.57°). Here, alanine exhibited a high hydrophobic nature compared to other amino acids complexed NiO, which was likely attributable to the better interaction of alanine with NiO, to have a higher nitrogen presence.^[^ ^82^, ^83^, ^84^ ^]^ It has been observed that, as the wettability of HTL decreases, perovskite nucleation sites are reduced and surface tension exerts a lesser force on the perovskite film, which promotes crystal growth and large particle size.^[^ ^85^, ^86^ ^]^ To investigate the characteristics of the substrate that affect the crystallinity of perovskite, X‐ray diffraction (XRD) investigations are performed as shown in Figure 4d. The samples have a typically orthorhombic perovskite phase, which is characterized by clear diffraction peaks at 14.1°, 24.4°, and 28.2°, and which correspond to the crystallographic planes of (100), (111), and (200), respectively. In contrast, the full width at half maximum (FWHM) value of the perovskite layer generated on top of the Ala complexed NiO was low, revealing the improved crystallinity and grain size in perovskite film.^[^ ^24^ ^]^ In addition, Atomic Force Microscopy (AFM) has been conducted to examine the effect of amino acid complexed NiO on the surface properties of the perovskite layer, as shown in Figure S8 (Supporting Information). The topographic image reveals that the root‐mean‐square (RMS) value of Ala complexed NiO‐based perovskite film is 10.19 nm, which is smaller than pristine (16.06 nm), Gly complexed (13.38 nm), and ABA complexed NiO (12.91 nm) based perovskite films, indicating that the Ala complexed NiO based perovskite is smoother, which is more beneficial to increase ohmic contact between layers and then improve device performance.^[^ ^87^ ^]^ The results presented above demonstrate that the Ala complexed NiO can enhance the growth of perovskite, which leads to larger grains, fewer grain boundaries, and a more crystalline perovskite film, which in turn can reduce the number of defect sites where charges can recombine and help improve the performance of the device.^[^ ^88^, ^89^ ^]^

**Figure 5:**
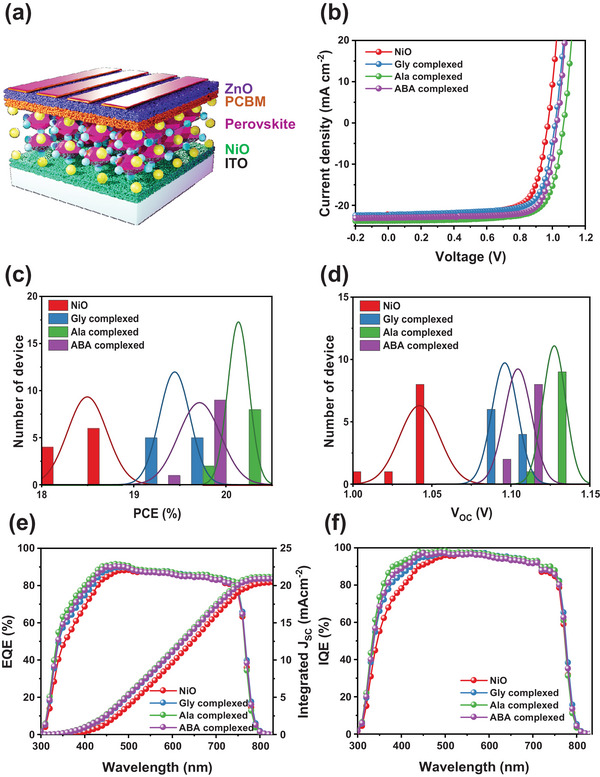
a) Schematic device architecture of p‐i‐n PSC with amino acid complexed NiO, b) current–voltage (*J–V*) curves, corresponding statistical histogram of c) PCEs, and d) V_OC_, e) external and f) internal quantum efficiencies of NiO and amino acid complexed NiO.

**Figure 6:**
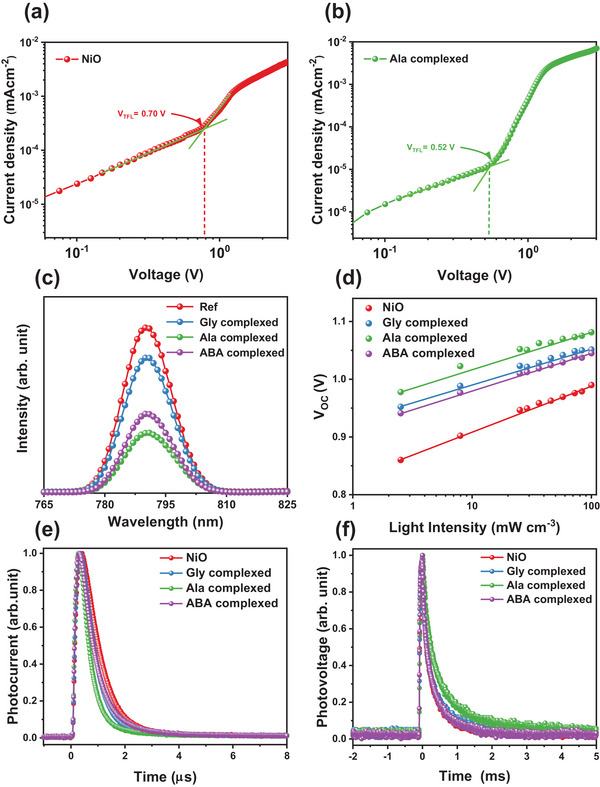
SCLC spectra of the a) NiO and b) Ala complexed NiO, c) PL spectra, d) light‐intensity dependence on V_OC_, e) transient photocurrent, and f) transient photovoltage of the NiO and amino acid complexed NiO.

To investigate the impact of amino acid‐based complexes on the overall efficiency of photovoltaic systems, p‐i‐n structured perovskite solar cells (PSCs) were assembled using the following device configuration: ITO/NiO complex/perovskite/PCBM/ZnO/Ag, as presented in **Figure** **5**a. Figures S9–S11 (Supporting Information) demonstrate that the use of 4, 4, and 2 mg concentrations of glycine, alanine, and aminobutyric acids resulted in improved mean and greatest efficiency values for PSCs compared to both the control and PSCs at various concentrations. Further experiments were performed based on these optimized concentrations of the amino acid material. Figure 5b displays the current–voltage (*J–V*) characteristics of the most optimal devices when subjected to AM 1.5 G illumination with an intensity of 100 mW cm^−2^. The devices with pristine NiO showed device performances with a V_OC_ of 1.05 V, a J_SC_ of 21.97 mA cm^−2^, FF of 78.85, and a PCE of 18.45%. On the other hand, the Gly complexed NiO device showed PCE of 19.59% with a V_OC_ of 1.10 V, J_SC_ of 22.05 mA cm^−2^, and FF of 80.04%, while the device with ABA complexed NiO showed an improved PCE of 19.92% with a V_OC_ of 1.11 V, J_SC_ of 22.27 mA cm^−2^, and FF of 79.98%. Meanwhile, the Ala complexed NiO device produced the best cell performance of 20.27% with a V_OC_ of 1.13 V, J_SC_ of 22.37 mA cm^−2^, and a FF of 80.15%. Figure 5c,d and Figure S12 (Supporting Information) illustrate histograms of the photovoltaic characteristics of the devices containing the pure NiO and amino acid complexed NiO. In addition, Ala complexed NiO devices showed minor hysteresis with a lower hysteresis index (HI) of 0.16 compared to 0.21 for the pristine PSCs, shown in Figure S13 (Supporting Information). The probable cause for this phenomenon is the increased extraction of charges at the interface between the hole transport layer (HTL) and the perovskite material, and the enhanced transportation of charge in the HTL.^[^ ^90^ ^]^ In addition, the external quantum efficiency (EQE) observations, as shown in Figure 5e, also support these improvements in photovoltaic properties. These interfacial modified PSCs exhibit greater photon‐to‐current conversion efficiencies across the board, as evidenced by their EQE spectra, compared to the pure NiO‐based PSCs. In addition, the J_SC_ value calculated from the EQE data of pristine NiO, Gly, Ala, and ABA complexed NiO‐based PSCs are 20.46, 21.00, 21.15, and 20.91 mA cm^−2^, respectively, as shown in **Figure** **6**d, and these calculated *J*_SC_ values are quite similar to the *J–V* curve based JSC within ≈5% error range. The measured internal quantum efficiency (IQE) for modified PSCs was more significant across the overall spectra compared to that of unmodified PSCs, as shown in Figure 5f. Specifically, the EQE and IQE improvement ratios of Ala complexed NiO‐modified PSCs exhibited comparable values, indicating that the PCE increases gained by the interfacial modifications were mainly attributable to evolution in charge extraction and transport abilities.^[^ ^24^, ^47^ ^]^ These observations indicate that Ala complexed NiO has improved capacities to extract and transport charges that are attributable to a reduction in surface defects, a larger grain size, improved crystallization, and a well‐aligned energy level.^[^ ^21^, ^80^ ^]^

For a more comprehensive study, devices consisting of an ITO/HTL (NiO, Ala complexed NiO/perovskite/PTAA/Ag) configuration are evaluated under dark conditions to measure the trap‐state density (N_trap_) of perovskite films using the space‐charge‐limited‐current (SCLC) strategy. Figure 6a,b and Figure S14 (Supporting Information) illustrate the gradual reduction of the trap‐filled limit voltage (V_TFL_) in the following order: control, Gly, ABA, and Ala complexed NiO devices. This reduction may be explained by the following equation:^[^ ^91^ ^]^

(3) $$N_{trap}=\frac{2\varepsilon_{0}\varepsilon V_{TFL}}{qL^{2}}$$

where the N_trap_ values for control Gly, Ala, and ABA complexed NiO devices are determined as 1.39 × 10^15^, 1.25 × 10^15^, 1.03 × 10^15^, and 1.15 × 10^15^ cm^−3^, respectively. The reduced defect density in the Ala complexed NiO sample can be attributed to the enhanced passivation of interfacial defects with the complexation process, as well as the improved quality of perovskite crystals.^[^ ^92^ ^]^ Moreover, the steady‐state photoluminescence (PL) measurements were conducted to examine and contrast the effects of the amino acid complexed NiO. The results shown in Figure 6c demonstrate that the amino acid complexed NiO/perovskite film exhibited superior PL quenching compared to the NiO/perovskite layer, and Ala complexed NiO HTL has enhanced hole extraction and transport capabilities compared to the unmodified NiO and other amino acid complexed NiO HTL, which can be attributed to the better energy band alignment and suppression of the redox reaction.^[^ ^93^, ^94^ ^]^ Further, considering that all electron‐hole pairs formed by light would ultimately recombine in the absence of an external circuit, the relationship between the open‐circuit voltage (V_OC_) and the intensity of illumination (I) can also elucidate the trap‐assisted recombination mechanism. The relationship between V_OC_ and I can be accurately modeled using the following linear equation:^[^ ^95^ ^]^

(4) $$V_{OC}=\frac{nK_{B}TIn\;\left(I\right)}{q}$$

where the variable n is the ideal factor used to assess the prevailing recombination process. Meanwhile, K_B_, T, and q correspond to the Boltzmann constant, absolute temperature, and elementary charge, respectively. The number of n is equal to 1, it represents bimolecular recombination, and when *n* is equal to 2, it represents Shockley‐Read‐Hall (SRH) recombination.^[^ ^96^ ^]^ The decreasing ideal factor in the pristine NiO (1.44), Gly complexed NiO (1.05), Ala complexed NiO (1.01), and ABA complexed NiO (1.07) devices indicates an increasing suppressive impact on trap‐assisted recombination, which aligns with the rising trend of V_OC_ (Figure 6d).^[^ ^95^ ^]^ Further, the utilization of transient photocurrent (TPC) and transient photovoltage (TPV) investigations provides a more thorough elucidation of the mechanisms involved in charge transport and recombination across the whole device. Figure 6e illustrates the decay durations of the photocurrent in NiO, Gly, Ala, and ABA complexed NiO PSCs, which are 0.72, 0.58, 0.46, and 0.64 µs, respectively. The shortened charge transport lifespan may be ascribed to improvements in the grain size, crystallization, and built‐in potential in the perovskite film, which augment the charge mobility and carrier extraction.^[^ ^97^ ^]^ Additionally, the photovoltage decay durations of NiO, Gly, Ala, and ABA complexed NiO PSCs are 0.23, 0.48, 0.61, and 0.56 ms, respectively, as shown in Figure 6f. The enhanced charge recombination lifespan potentially indicates that the Ala complexed NiO successfully decreases the density of surface defects and prevents charge recombination in the perovskite layer.^[^ ^98^ ^]^ These observations indicate that the Ala complexed NiO can lower the number of defects, enhance the efficiency of extracting and transporting charge carriers, and minimize recombination assisted by traps due to the better energy band alignment, large grain size, and suppressed interfacial redox reaction, which can lead to overall improvements in V_OC_, J_SC_, and FF.^[^ ^18^, ^99^ ^]^

**Figure 7:**
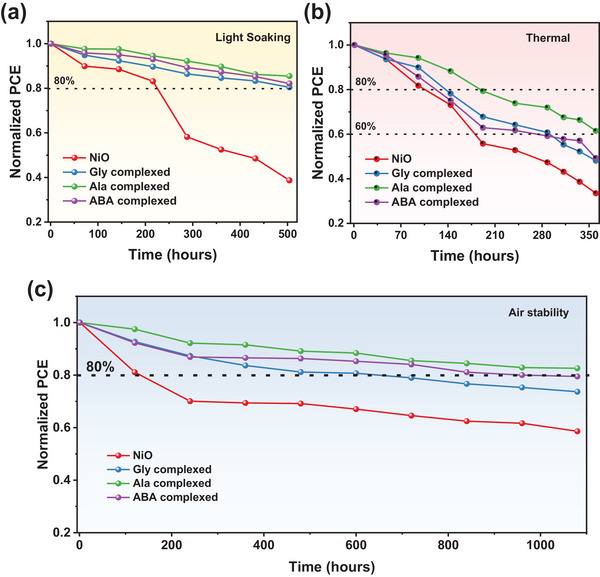
a) Light soaking stability measured under constant light illumination, b) thermal stability measured under heating at 85 °C, and c) normalized PCE as a function of time in air ambient conditions of NiO and amino acid complexed NiO.

The long‐term stability of PSCs is a crucial factor that has to be addressed to achieve commercialization. Herein, we performed stability studies to verify the reliability of PSCs fabricated with an amino acid complexed NiO, while considering the impacts of light, moisture, oxygen, and heat on the function of the device for a longer duration without encapsulation. Under constant illumination, the maximum power point voltage and PCE performance of the devices are displayed in Figure S15 (Supporting Information). We discovered the steady‐state photocurrent outputs of all four devices (unmodified NiO layer and the Gly, Ala, and ABA complexed NiO) are very stable. Around 1800 s was needed for the Ala complexed NiO‐based devices to reach steady‐state photocurrent density (22.31 mA cm^−2^) and PCE (20.12%). In addition, there was a notable disparity in the extended‐term operational durability of the devices between the unencapsulated pristine and Gly, Ala, and ABA complexed NiO PSCs (**Figure** **7**a). The efficiency of Ala complexed NiO PSCs remains ≈85% after 500 h, indicating long‐term light stability; nonetheless, under the same conditions, pure NiO‐based PSCs preserve 38% efficiency, Gly complexed NiO PSCs retain 80% efficiency, and ABA complexed NiO PSCs retain 82% efficiency. Moreover, the thermal stability of devices based on NiO, Gly, Ala, and ABA complexed NiO PSCs was studied in a nitrogen‐filled glove box that had been heated to 85 °C for 350 h (Figure 7b). The Gly, Ala, and ABA complexed NiO PSCs maintained 48%, 61%, and 49% of their original efficiencies, respectively, after being stored on a hot plate at a temperature of 85 °C for a duration of 350 h in a dark glovebox filled with nitrogen gas. In contrast, pristine NiO PSCs only maintained 33% of their original efficiency after encountering the same circumstances. Moreover, to determine their functional durability in the presence of oxygen and moisture, unencapsulated PSCs were subjected to a 1000‐h test at room temperature and humidity (25–30 °C, 45–55% relative humidity), as illustrated in Figure 7c. The air stability of Ala complexed NiO PSCs was shown to be significantly improved, with a retention of 82% of the original efficiency over 1000 h at a relative humidity (RH) of 20–30% at room temperature, compared to the NiO device, Gly, and ABA complexed NiO PSCs, which maintained 58%, 73%, and 79% efficiency, respectively. The findings indicate that the devices with Ala complexed NiO exhibited significant improvements in their resistance to oxygen, moisture, light, and heat after optimization.

To further confirm the interaction between the HTL and the perovskite layer precursor under harsh conditions, X‐ray diffraction (XRD) spectroscopy was performed after 24 and 48 h while maintaining constant heat environments. The pure NiO shows the PbI_2‐x_Br_x_ phase (with a peak at 12.8).^[^ ^62^ ^]^ The quick decrease in performance observed after 24 h is considered to be due to the absence of an A‐site cation.^[^ ^19^ ^]^ The perovskite layer undergoes distortion when the PbI_2‐x_Br_x_ peak emerges, and the absence of A‐site cations in the perovskite layer impacts the crystal structure, as shown in Figure S16a (Supporting Information).^[^ ^26^, ^76^ ^]^ On the other hand, the X‐ray diffraction (XRD) pattern of the Gly, Ala, and ABA complexed NiO PSCs did not exhibit any indications of the PbI_2‐x_Br_x_ phase. However, the PbI_2‐x_Br_x_ phase appeared in the Gly and ABA complexed NiO PSCs after being heated at 85 °C for 48 h, as shown in Figure S16b (Supporting Information), while Ala complexed NiO PSCs did not show the presence of the PbI_2‐x_Br_x_ phase. These results suggest that alanine can show a stronger interaction with NiO than the other amino acid complexed NiO. This can be attributed to the fact that even‐numbered n‐alkanes in alanine facilitate favorable intermolecular interactions at both ends, owing to their symmetrical arrangements of carbon atoms.^[^ ^100^, ^101^ ^]^ This symmetry allows for a balanced distribution of Van der Waals forces, ensuring optimal interaction potential at both ends of the molecule. On the other hand, odd‐numbered n‐alkanes in glycine and amino butyric acid lack this symmetrical disposition, which results in mainly concentrated intermolecular interactions at one end. Consequently, the other end of the molecule experiences relatively greater distances between atoms, which leads to a less favorable interaction environment.^[^ ^102^ ^]^ As a result, an even‐numbered alanine can pack more efficiently into ordered periodic crystalline structures, and exhibit higher densities, more symmetrical arrangements, and closer packing in the crystal lattice. These factors contribute to stronger intermolecular forces and greater bonding strength in alanine (with an even alkane number) compared to glycine and aminobutyric acid (with odd alkane numbers), which results in a more promising and stronger interaction of the alanine with NiO compared to both other amino acids.^[^ ^100^, ^101^, ^102^ ^]^ These findings indicate that the Ala complexed NiO functions as a rehabilitative agent that effectively reduces defects such as NiOOH species on NiO surfaces, which regenerates the NiO‐perovskite interface by preventing the formation of PbI_2‐x_Br_x_.^[^ ^18^, ^62^ ^]^ These observations suggest that the introduction of an Ala complexed NiO can effectively enhance the quality of perovskite films, with the dual purpose of passivating interface defects as well as eliminating residual strain and redox reactions at the NiO/perovskite interface, which leads to a significant improvement in the long‐term stability of PSCs.^[^ ^18^, ^19^ ^]^

**Figure 8:**
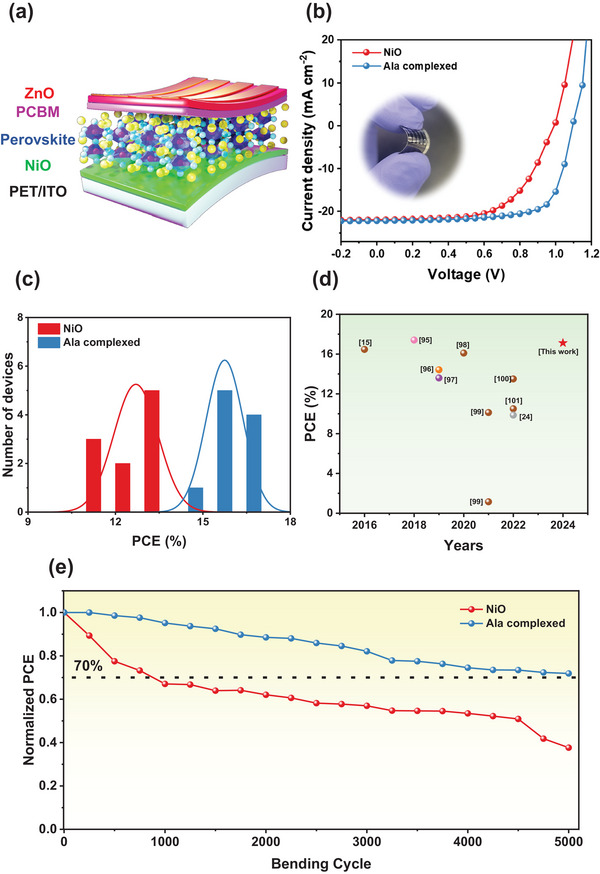
a) Schematic illustration of flexible PSCs, b) current–voltage (*J–V*) curves, c) corresponding statistical histogram of F‐PSCs, d) power conversion efficiency (PCE) of F‐PSCs as a function of years from both this work and recent representative reports, and e) normalized PCE as a function of cyclic bending test parameters of NiO and Ala complexed NiO F‐PSCs.

To illustrate the significance of our research, Table S1 (Supporting Information) compares our present work with previous studies on perovskite solar cells that utilized various dopants and composites for NiO HTM. However, only a few studies have shown the deposition of NiO‐based HTL film at room temperature, which needs to undergo high‐temperature annealing (ranging from 120 to 500 °C) to form NiO.^[^ ^51^ ^]^ However, it is essential to refrain from using high‐temperature procedures while working with flexible PSC devices. Consequently, it is crucial to investigate the low‐temperature operations of HTLs. Room temperature processing is crucial for both simplifying the fabrication processes and facilitating production on flexible substrates. The HTL processing on flexible substrate is typically restricted to temperatures above 130 °C because of the low heat sensitivity of polymers.^[^ ^51^, ^56^ ^]^ To investigate the effects of temperatures (100, 130, 150, and 180 °C) on Ala complexed NiO, we conducted a comprehensive analysis of the optical, AFM, SKPM, and photovoltaic characteristics of the different temperature‐treated NiO and Ala complexed NiO. The results show no major change in the optical transmittances of the NiO and Ala complexed NiO after treatment at different temperatures of thin film, as presented in Figure S17 (Supporting Information), which indicates that the temperature does not lead to a change in the optical transmittance of NiO and Ala complexed NiO thin film. Further, to evaluate the impact of temperature on the surface characteristics of HTL, we conducted measurements of AFM and SKPM analysis of the NiO and Ala complexed NiO after treatment at different temperatures, as shown in Figures S18 and S19 (Supporting Information). The different temperature‐treated NiO showed a similar roughness; the root‐mean‐square roughness (rms) of pristine NiO treated at 100, 130, 150, and 180 °C was ≈9.36, 9.50, 9.20, and 9.45 nm, respectively. The root‐mean‐square roughness (rms) of the Ala complexed NiO samples treated at 100, 130, 150, and 180 °C were ≈5.62, 5.72, 5.75, and 5.35 nm, respectively. The Ala complexed NiO samples treated at different temperatures exhibited similar roughness values. Further, the different temperature‐treated NiO and Ala complexed NiO retain the same surface potential value, indicating that the different temperature‐treated NiO and Ala complexed NiO have similar work functions (WF). These results indicate the temperature did not have a significant impact on the surface and electronic properties of the NiO and Ala complexed NiO thin film. In addition, the photovoltaic properties of the various temperature‐treated NiO and Ala complexed NiO thin films were also tested to validate the effects of the different temperatures, and the results are presented in Figure S20 (Supporting Information). The performance of the various temperature‐treated NiO and Ala complexed NiO is quite similar compared to the pristine NiO and Ala complexed NiO treated PSCs at 100 °C, which showed a similar trend, as we discussed above. These findings showed that the optical, physical, and electrical characteristics of the NiO and Ala complexed NiO were unchanged by temperature and maintained a comparable PCE. Therefore, our study offers a more convenient method for constructing flexible electronics at low temperatures compared to other existing reports, as described in Table S1 (Supporting Information). Therefore, to analyze the significance of an Ala complexed NiO solution processable at room temperature as a hole transport layer (HTL), we manufactured PSCs on flexible ITO‐PET substrates. **Figure** **8**a displays a schematic representation of the flexible PSCs (F‐PSCs) architecture. Figure 8b displays the *J–V* curves of the F‐PSCs utilizing pure NiO and Ala complexed NiO as HTL and Figure 8b (inset image) shows an image of the equivalent typical flexible device. The pristine NiO device has a PCE of 13.54%, a V_OC_ of 0.97 V, a J_SC_ of 21.68 mA cm^−2^, and an FF of 63.58%. Meanwhile, the Ala complexed NiO device obtained the best cell performance of 17.12% with a V_OC_ of 1.05 V, J_SC_ of 22.01 mA cm^−2^, and a FF of 74.04%. The photovoltaic properties of devices containing pure NiO and Ala complexed NiO are shown as a series of histograms in Figure 8c. While the efficiency of flexible devices is somewhat lower than that of PSCs on ITO‐glass substrates, this is possibly attributable to the fact that ITO‐PET flexible substrates have a greater sheet resistance (∼60 Ω/sq) than ITO‐glass substrates (≈10 Ω sq^−1^).^[^ ^56^, ^103^ ^]^ Further, the PCE value is similarly equivalent to that of identical NiO‐based F‐PSCs fabricated using alternative techniques, as in shown Figure 8d.^[^ ^16^, ^25^, ^52^, ^104^, ^105^, ^106^, ^107^, ^108^, ^109^ ^]^ Moreover, the mechanical durability of flexible perovskite devices deposited on low‐temperature NiO and Ala complexed NiO F‐PSCs has been examined through repetitive bending of the whole device. Figure 8e displays the consequences of 5000 subsequent bending cycles on both types of NiO and Ala complexed NiO F‐PSCs with a curvature radius of 2 mm. After undergoing 5000 cycles with a curvature radius of 2 mm, the PCE of devices containing pristine NiO decreases to 37% of the original PCE. Nevertheless, the PCE of devices employing Ala complexed NiO HTL remains at 71% of its original value (as shown in Figure 8e). The enhanced mechanical stability associated with a narrow radius promotes the widespread use of wearable electronics. These results also validate that incorporating an Ala complexed NiO enhances the capacity of F‐PSCs to tolerate bending, particularly in narrow bending tests, which is likely attributable to its exceptional mechanical stability and better interfacial contact with the perovskite layer.^[^ ^26^, ^62^, ^110^, ^111^ ^]^ Overall, these observations establish that the Ala complexed NiO provided an innovative perspective on circumventing the redox reaction at the interface between NiO and perovskites and significantly enhancing the bending resistance of flexible PSCs, even in narrow bending tests; this is a critical impediment to the future development of wearable and stable photovoltaic devices.

## 3 Conclusion

In conclusion, this work utilized an amino acid complexed NiO as an interface modifier for the first time to eliminate hydroxyl groups and defect sites from the surface of NiO. Consequently, the amino acid complexed NiO effectively addresses many issues by passivating interfacial defects, suppressing nonradiative recombination, preventing undesired redox reactions, and relieving stress at the NiO/perovskite interface, which leads to a substantial enhancement in PCE of the devices. In addition, the amino acid complexed NiO also facilitated enhanced energy level alignment between the NiO HTL and the perovskite layer, thus leading to superior charge extraction and transport as well as decreased charge recombination. Further, the amino acid complexed NiO enhanced the crystal structure and grain size of the perovskite, which led to improved charge extraction and transportation. The device Ala complexed NiO achieves an impressive FF of 80.15%, a J_SC_ of 22.37 mA cm^−2^, a high V_OC_ of 1.13 V, and a champion PCE of 20.27%. Ala complexed NiO PSCs also exhibit improved stability while exposed to ambient air, continuous light irradiation, and heating to 85 °C, retaining over 82%, 85%, and 61% of their initial PCE after 1,000, 500, and 330 h, respectively. Moreover, the low‐temperature NiO deposition technique is favorable for fabricating flexible PSCs. Therefore, Ala‐complexed NiO flexible PSCs have been fabricated and attained an efficiency of 17.12% and maintained 71% of their initial PCE after undergoing 5 000 bending tests. It is anticipated that the current study will aid in the transition to perovskite photovoltaics by illuminating a prospective avenue for the development of high‐quality HTLs.

## 4 Experimental Section

The Experimental Section can be found in the Supporting Information.

## Conflict of Interest

The authors declare no conflict of interest.

## Supporting information



Supporting Information

## Data Availability

The data that support the findings of this study are available from the corresponding author upon reasonable request.
