# Supplementary material for: Interfacial Engineering of Nickel Oxide‐Perovskite Interface with Amino Acid Complexed NiO to Improve Perovskite Solar Cell Performance
Source: Small. 2024 Sep 20;20(49):2405953. doi: 10.1002/smll.202405953 (PMC11618699; doi:10.1002/smll.202405953)
Supplement: Supplementary file 1 — Supporting Information [file SMLL-20-2405953-s001.docx]

*Supplementary Information*

**Interfacial Engineering of Nickel Oxide-Perovskite Interface with Amino Acid Complexed NiO to Improve Perovskite Solar Cell Performance**

Dilpreet Singh Mann ^a^, Sakshi Thakur ^a^, Sushil S. Sangale ^a^, Kwang-Un Jeong ^b^ Sung-Nam Kwon ^a^, and Seok-In Na ^a^*

^a^ Department of Flexible and Printable Electronics and LANL-JBNU Engineering Institute-Korea, Jeonbuk National University, 567, Baekje-daero, Deokjin-gu, Jeonju-si, 54896, Republic of Korea

^b^ Department of Polymer-Nano Science and Technology, Department of Nano Convergence Engineering, Jeonbuk National University, 567, Baekje-daero, Deokjin-gu, Jeonju-si, 54896, Republic of Korea

-------------------------------

* Corresponding author. Tel.: +82 63 270 4465; fax: +82 63 270 2341

E-mail address: nsi@jbnu.ac.kr (Prof. S.-I. Na)

**Experimental procedure**

*Materials:* The following materials were purchased from Sigma-Aldrich: Nickel Nitrate, cesium iodide (CsI), Glycine, Alanine, Amino butyric acid, N, N-dimethyl-formamide (DMF), dimethyl sulfoxide (DMSO), and chlorobenzene (CB). Great Cell Solar provided Formamidine iodide (FAI) and methylammonium bromide (MABr). This study employed lead iodide (PbI2) and lead bromide (PbBr) provided by Tokyo Chemical Industry. Nano Clean Tech provided a suspension of zinc oxide nanoparticles (ZnO) in 2-propanol. Nano C supplied PCBM, which stands for Phenyl-C61-butyric acid methyl ester. All chemicals were used in their crude state for the experiment.

*Synthesis of pristine NiO and complexed NiO:* A chemical precipitation method was used to make pure NiO NPs [1, 2]. Ni (NO_3_)_2_·6H_2_O (3 g) was mixed with 60 ml of deionized (DI) water and stirred constantly at room temperature to achieve a clear green solution. The ammonium hydroxide (NH_4_OH) solution was added slowly while stirring until the pH reached 10 and stirring continued for another 10 minutes. The green colloidal material was obtained by centrifuging at 10,000 rpm for 15 minutes and then washed twice with DI water and ethanol. The green adduct was dried in an 80°C hot air oven overnight, ground up, and then heated to 270°C for two hours to make a dark-black powder. Afterward, to prepare the solution of NiO complexed with amino acids, various concentrations of amino acids such as glycine, alanine, and aminobutyric acid (2, 4, 6, 8, and 10 mmol) were added to a dispersion of NiO nanoparticles. Lastly, the mixed dispersion solution was stirred for 24 hours. The prepared amino acid complexed NiO solution was directly used as an HTL for perovskite solar cells.

*Device fabrication:* The preparation processes for ITO, NiO, perovskite, PCBM, ZnO, and Ag were the same as those previously described in our research [3]. The thin films NiO and amine based NiO complex were coated for 30 seconds at a speed of 3000 revolutions per minute (rpm) and heated at a temperature of 100°C for a period of 10 minutes. The thin perovskite films were coated using the spin-coating technique. In the first step, the films were coated for 5 seconds at a speed of 500 rpm. In the second step, the coating process continued for 45 seconds at a speed of 4500 rpm. Prior to the coating procedure, the amount of 1,2-Dichlorobenzene (DCB) anti-solvent was gradually reduced starting from 30 seconds. After the coating, the films were annealed at a temperature of 100°C for a duration of 30 minutes. PCBM was mixed with anhydrous DCB and applied onto perovskite films by spinning at a speed of 6000 rpm for 50 seconds. Subsequently, the films were heated at a temperature of 100°C for 10 minutes. The PCBM layer was coated with ZnO nanoparticles using a two-step process: first, for 15 seconds at a speed of 1000 rpm, and then for 40 seconds at a speed of 8000 rpm. Subsequently, a period of 10 minutes of annealing at a temperature of 100°C was carried out.

*Characterizations:* The application of X-ray photoelectron spectroscopy, provided by Thermo Fischer Scientific, was employed to determine the elemental composition of both NiO and amino acids complexed NiO PSCs. The surface topography image of perovskite layers and elemental mapping were obtained using scanning electron microscopy (SEM) with a Carl Zeiss-SupraTM-40VP microscope. An XE7 Park Systems was used to evaluate the surface potential and roughness of NiO and amino acids complexed NiO PSCs during atomic force microscopy. The crystal structure of perovskite films was analyzed using a Rigaku Ultima X-ray diffractometer (XRD). Analyzing properties of the device, such as current density-voltage (J-V), was done using an Oriel Sol3A solar simulator and a Keithley 2400 source meter. The light intensity condition of 1-sun (100 mW/cm^2^) was calibrated by employing a conventional crystalline Si cell. The photon-to-electron conversion efficiency was thoroughly investigated using an IQE-200 measuring device manufactured by Oriel Instruments in the United States. The measurement of photoluminescence (PL) was conducted using a Shimadzu Lab Solutions RF spectrofluorometer. The McScience T4000 measurement equipment was used for the investigation of transient photocurrent (TPC) and transient photovoltage (TPV). The constant light illumination measurement was done using the Newport LSH-7320 LED solar simulator. The light intensity condition of 1-sun (100 mW/cm^2^) was calibrated by employing a conventional crystalline Si cell.

**Fig. S1.** (a) XRD spectra of NiO powder and (b) image of NiO dispersion in DI water solution at fresh and after 30 days.

**Fig. S2.** (a) Transmission spectra and (b) Tauc plot of NiO thin film.

**Figure. S3**. Relative ratio of (a) NiOOH, (b) Ni^3+^/Ni^2+^ species obtained from peak fitting of Ni 2p and O 1s core level spectra of XPS, and percentage of the (c) nitrogen of NiO and amino acid complexed NiO thin film.

**Figure S4.** SEM images of NiO and amino acid complexed NiO thin film

**Figure. S5.** (a) UPS spectra for Gly and ABA complexed NiO thin films and (b) schematic diagram of energy level alignment of PSCs with NiO and Ala complexed NiO.

**Fig. S6.** SEM images of perovskite films fabricated onto the Gly, and ABA complexed NiO thin films.

**Fig. S****7.** Contact angle (CA) of the NiO and amino acid complexed NiO

**Figure S8.** AFM images of perovskite films fabricated onto NiO and amino acid complexed NiO.

**Fig. S9.** Current density-voltage (J-V) curves and corresponding statistics of power conversion efficiency (PCE) of pristine and Gly complexed NiO PSCs.

**Fig. S10.** Current density-voltage (J-V) curves and corresponding statistics of power conversion efficiency (PCE) of pristine and Ala complexed NiO PSCs.

**Fig. S11.** Current density-voltage (J-V) curves and corresponding statistics of power conversion efficiency (PCE) of pristine and ABA complexed NiO PSCs.

**Fig. S12.** Corresponding statistical histogram of (a) J_SC_, and (b) FF of the NiO and amino acid complexed NiO.

**Figure S13.** *J–V* curves for NiO and amino acid complexed NiO based device under forward and reverse scan directions. The hysteresis index can be calculated through the formula:$HI=\frac{{PCE}_{Reverse}-{PCE}_{Forward}}{{PCE}_{Reverse}}$.

**Fig. S14.** SCLC spectra of the (a) Gly and (b) ABA complexed NiO.


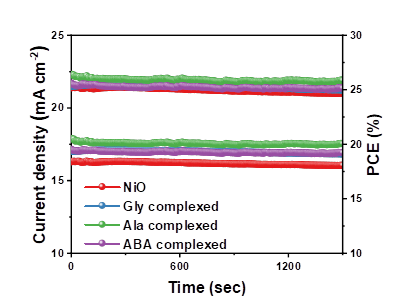


**Figure S15.** Steady-state photocurrent and PCE measured at maximum power point under constant light illumination of for NiO and amino acid complexed NiO based device.

**Fig. S16.** XRD spectra of perovskite with NiO and amino acid complexed NiO after (a) 24 and (b) 48 hours at 85℃.

**Fig. S17**. UV-visible spectra of (a) NiO and (b) Ala complexed NiO thin films after annealing at different temperatures.

**Fig. S18**. Atomic force microscopy (AFM) of (a) NiO and (b) Ala complexed NiO thin films after annealing at different temperatures.

**Fig. S19**. Scanning Kelvin probe microscopy (SKPM) of (a) NiO and (b) Ala complexed NiO thin films after annealing at different temperatures.

**Fig. S20**. Corresponding statistics of power conversion efficiency (PCE) of (a) NiO and (b) Ala complexed NiO thin films after annealing at different temperatures.

**Table S1**. Comparison table of Perovskite solar devices (rigid substrate/NiO) based on different NiO HTL.

| **Device structure** | **Synthesis method** | **Temperature** | **PCE (%)** | **Ref.** |
| --- | --- | --- | --- | --- |
| Cu:NiO | Sol-gel | 300 ℃ | 18.30 | [4] |
| Cu:NiO | Chemical Precipitation | 120 ℃ | 18.66 | [5] |
| Cu:NiO | Solution processed | 120 ℃ | 20.26 | [6] |
| Co:NiO | Solution processed | 150 ℃ | 18.60 | [7] |
| Cs:NiO | Sol-gel | 275 ℃ | 19.35 | [8] |
| Rb:NiO | Sol-gel | 200 ◦C | 17.21 | [9] |
| Zn:NiO | Sol-gel | 400 ℃ | 19.60 | [10] |
| Fe:NiO | Chemical Precipitation | 100 ℃ | 17.57 | [2] |
| N:NiO | Sol-gel | 280℃ | 17.02 | [11] |
| V:NiO | Solution processed | 100 ℃ | 15.45 | [1] |
| NiO:BN | Solution processed | 350 ℃ | 20.74 | [3] |
| NiO:N-RGO | Solution processed | 100 ℃ | 18.84 | [12] |
| Ala complexed NiO | Solution processed | 100 ℃ | 20.27 | This work |

**References**

1. Kotta, A.; Seo, I.; Shin, H.-S.; Seo, H.-K., *Chemical Engineering Journal* **2022,** *435*, 134805.

2. Chandrasekhar, P.; Seo, Y.-H.; Noh, Y.-J.; Na, S.-I., *Applied Surface Science* **2019,** *481*, 588-596.

3. Mann, D. S.; Patil, P.; Kim, D.-H.; Kwon, S.-N.; Na, S.-I., *Journal of Power Sources* **2020,** *477*, 228738.

4. Zhang, C.; Li, W.; Liu, C.; Fan, J.; Mai, Y., *Solar RRL* **2017,** *1* (10), 1700141.

5. He, Q.; Yao, K.; Wang, X.; Xia, X.; Leng, S.; Li, F., *ACS applied materials & interfaces* **2017,** *9* (48), 41887-41897.

6. Chen, W.; Wu, Y.; Fan, J.; Djurišić, A. B.; Liu, F.; Tam, H. W.; Ng, A.; Surya, C.; Chan, W. K.; Wang, D., *Advanced Energy Materials* **2018,** *8* (19), 1703519.

7. Xie, Y.; Lu, K.; Duan, J.; Jiang, Y.; Hu, L.; Liu, T.; Zhou, Y.; Hu, B., *ACS applied materials & interfaces* **2018,** *10* (16), 14153-14159.

8. Chen, W.; Liu, F. Z.; Feng, X. Y.; Djurišić, A. B.; Chan, W. K.; He, Z. B., *Advanced Energy Materials* **2017,** *7* (19), 1700722.

9. Fu, Q.; Xiao, S.; Tang, X.; Hu, T., *Organic Electronics* **2019,** *69*, 34-41.

10. Wan, X.; Jiang, Y.; Qiu, Z.; Zhang, H.; Zhu, X.; Sikandar, I.; Liu, X.; Chen, X.; Cao, B., *ACS Applied Energy Materials* **2018,** *1* (8), 3947-3954.

11. Zhou, P.; Li, B.; Fang, Z.; Zhou, W.; Zhang, M.; Hu, W.; Chen, T.; Xiao, Z.; Yang, S., *Solar RRL* **2019,** *3* (10), 1900164.

12. Zhang, F.; Mezgeb, M. M.; Guo, W.; Xu, H.; Liu, X.-Y., *Journal of Power Sources* **2018,** *399*, 246-253.
